# Inhibitory Effects of Resveratrol on the Epstein-Barr Virus Lytic Cycle 

**DOI:** 10.3390/molecules15107115

**Published:** 2010-10-14

**Authors:** Ching-Yi Yiu, Shih-Ying Chen, Li-Kwan Chang, Ya-Fang Chiu, Tsuey-Pin Lin

**Affiliations:** 1 Department of Otolaryngology, Chi Mei Medical Center, Liouying; Tainan, 717, Taiwan; 2 Department of Applied Life Science and Health, Chia-Nan University of Pharmacy and Science, 60 Erh-Jen Road, Sec. 1, Jen-Te Hsiang, Tainan, 717, Taiwan; 3 Institute of Microbiology and Biochemistry, National Taiwan University, Taipei, 106, Taiwan; 4 Molecular Genetics Laboratory, Department of Microbiology and Immunology, Chang-Gung University, Taoyuan, 333, Taiwan; 5 Department of Health and Nutrition, Chia-Nan University of Pharmacy and Science, 60 Erh-Jen Road, Sec. 1, Jen-Te Hsiang, Tainan, 717, Taiwan

**Keywords:** resveratrol, antiviral activity, EBV

## Abstract

Reactivation of Epstein-Barr virus (EBV) from latency to the lytic cycle is required for the production of viral particles. Here, we examine the capacity of resveratrol to inhibit the EBV lytic cycle. Our results show that resveratrol inhibits the transcription of EBV immediate early genes, the expression of EBV lytic proteins, including Rta, Zta, and EA-D and reduces viron production, suggesting that this compound may be useful for preventing the proliferation of the virus.

## 1. Introduction

Epstein-Barr virus (EBV) is a gamma-herpesvirus, which infects human lymphoid cells and epithelial cells [1]. Infection by this virus is associated with a number of human cancers, including Burkitt’s lymphoma [2], nasopharyngeal carcinoma (NPC) [3] and Hodgkin’s disease [4]. The reactivation of EBV from latency to the lytic cycle is necessary for the virus to produce virions and establish infections [5,6]. At the onset of the lytic cycle, EBV expresses two transcription factors, Rta and Zta, which are transcribed from BRLF1 and BZLF1, respectively [7]. These two proteins trigger an ordered cascade of the expression of viral lytic genes, including that of BMRF1 and BALF5, which encode diffused early antigen (EA-D) and DNA polymerase [8]. Rta and Zta can also activate IL-6 transcription in lytically-infected B cells, which leads to immortalized B cells [9]. Therefore, to reduce the disease risk and to improve the clinical outcome an effective strategy to block the viral lytic cycle is of value. Earlier studies have established that lytic EBV replication is inhibited by acyclovir and ganciclovir, which specifically inhibit the function of viral-encoded DNA polymerase [10]. An earlier study showed that epigallocatechin gallate (EGCG) inhibits the expression of the EBV immediate-early genes transcription at concentration 50 μM [11]. Ethanolic extract from *Andrographis paniculata* and andrograpolide also inhibit the expression of Rta, Zta and EA-D at 25 μg/mL and 5 μg/mL, respectively [12]. 

Resveratrol is a non-flavonoid polyphenol compound present in many plants and fruits. This compound has a high bioactivity and its cytoprotective action has been demonstrated. Previous studies showed that resveratrol inhibits the replication of human cytomegalovirus (HCMV) [13], herpes simplex virus type 1 (HSV-1) [14] and Varicella-zoster virus (VZV) [15]. Furthermore, resveratrol also has been reported to inhibit the expression of EBV early antigen induced by 12-*O*- tetradecanoylphorbol 13-acetate (TPA) in Raji cells [16]. However, no preexisting study has been reported on resveratrol mediated the inhibition of EBV lytic cycle. Thus, the purpose of our study was to evaluate whether resveratrol inhibits the transcription of EBV immediate early genes, the expression of EBV lytic proteins, including Rta, Zta, and EA-D and viron production.

## 2. Results and Discussion

### 2.1. Determining the toxicity of resveratrol to P3HR1 cells

**Figure 1:**
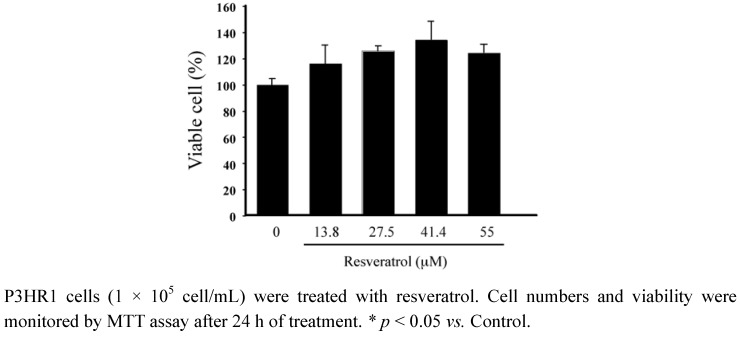
Effect of resveratrol on cell viability.

Resveratrol was added into 1 × 10^5^ cells/mL P3HR1 cells. After 24 h of treatment, cell viability was determined by the MTT assay. Results showed that resveratrol exhibited no cytotoxicity toward P3HR1 cells at concentrations below 55 μM (Figure 1). 

### 2.2. Resveratrol inhibit the expression of viral lytic proteins

**Figure 2:**
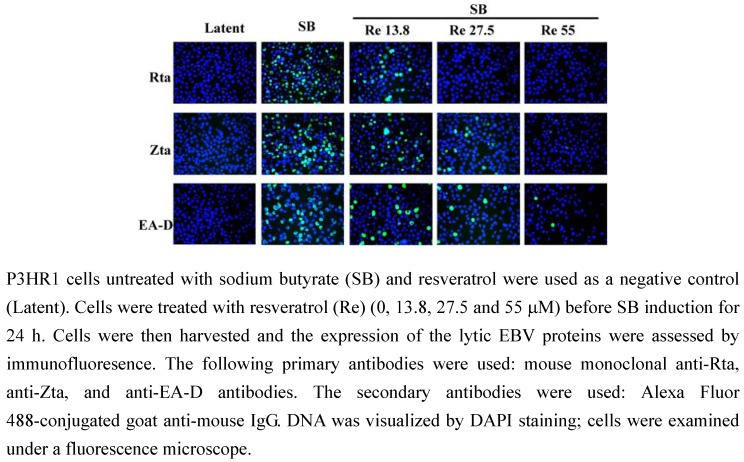
Indriect immunofluorescence analysis of inhibition effects on the expression of EBV lytic genes.

Resveratrol was added to P3HR1 cells (3 × 10^6^) that had been treated with sodium butyrate to activate the EBV lytic cycle. At 24 h after lytic induction, the expression of Rta, Zta and EA-D was examined by an immunofluorescence assay which showed that at 13.8 μM, resveratrol reduced the expression of Rta, Zta and EA-D (Figure 2). At 27.5 μM, the expression of Zta and EA-D was substantially reduced and no expression of Rta was observed (Figure 2). When the concentration of resveratrol increased to 55 μM, no expression of Rta and Zta was observed and only few cells expressed EA-D (Figure 2).

### 2.3. Flow cytometric analysis of the expression of EBV lytic proteins

**Figure 3:**
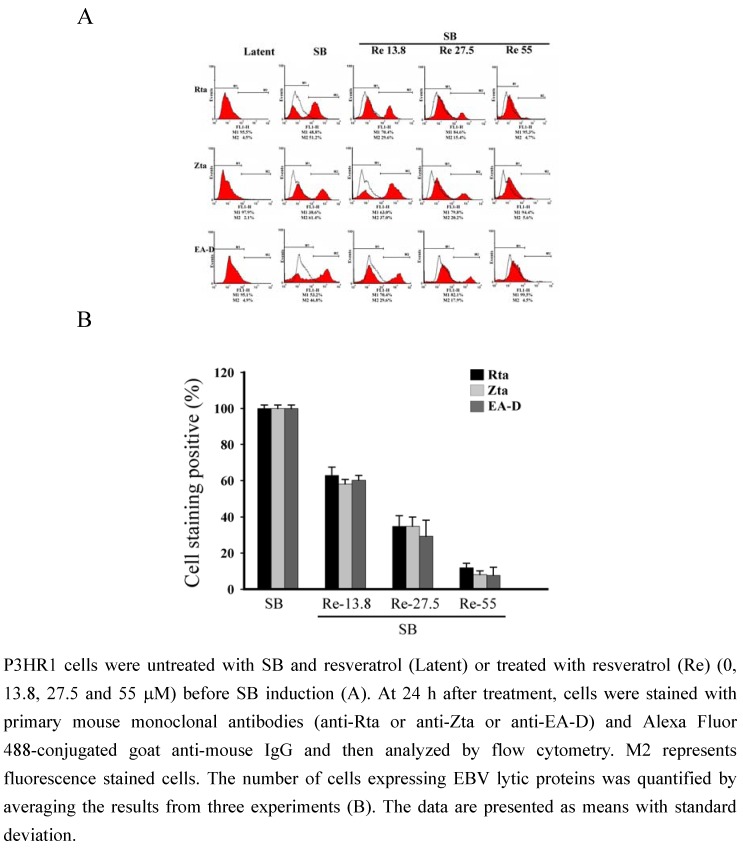
Inhibition of the expression of EBV lytic proteins by resveratrol.

The presence of EBV lytic proteins in P3HR1 cells was further analyzed by flow cytometric analysis. The P3HR1 populations not treated with SB that expressed Rta, Zta and EA-D wwere 4.5%, 2.1% and 4.9%, respectively (Figure 3). The populations that expressed the three proteins after SB treatment increased to 51.2%, 61.4% and 46.8%, respectively. After treating the cells with 13.8 μM of resveratrol, the populations that expressed Rta, Zta and EA-D decreased to 29.6%, 37.0% and 29.6%, respectively (Figure 3A The populations that expressed Rta, Zta, and EA-D further decreased when the concentration of resveratrol was increased to 27.5 μM and 55 μM. At 27.5 μM, the populations that expressed Rta, Zta, and EA-D decreased to 15.4%, 20.2%, and 17.9%, respectively (Figure 3). At 55 μM, the population that expressed Rta was reduced from 51.2% to 4.7% (Figure 3); the population that expressed Zta, from 61.4% to 5.6%; and the population that expressed EA-D from 46.8% to 4.5%, respectively (Figure 3). These results show that resveratrol inhibited the expression of Rta, Zta and EA-D in a dose-dependent manner (Figure 3B).

### 2.4. Inhibiting the transcription of EBV immediate-early genes

**Figure 4:**
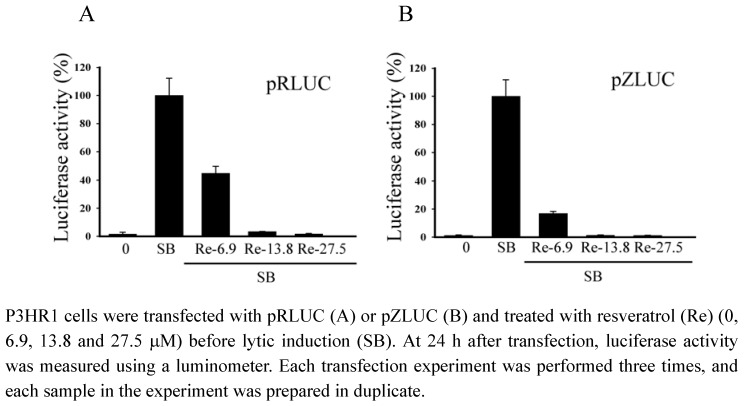
Inhibition of the transcription of the BRLF1 and BZLF1 promoters by resveratrol.

The activity of the BRLF1 and BZLF1 promoters was analyzed by transient transfection assay in P3HR1 cells using the reporter plasmids pRLUC and pZLUC, respectively. Results showed that resveratrol at 6.9 μM inhibited the activity of BRLF1 and BZLF1 promoters by 56% and 83%. Resveratrol at 13.8 μM decreased the BRLF1 and BZLF1 promoters’ activity to background (Figure 4).

### 2.5. Inhibition of the production of EBV particles

**Figure 5:**
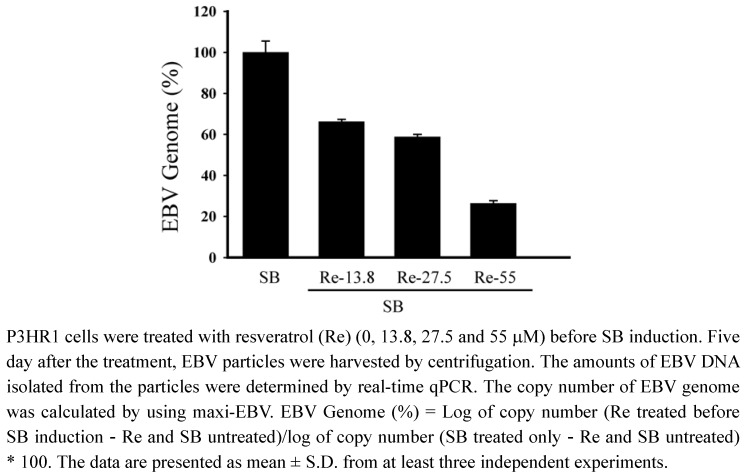
Resveratrol treatment and production of EBV particles.

P3HR1 cells were then treated with 13.8, 27.5 and 55 μM of resveratrol after lytic induction. After culturing for five days, EBV particles released into medium were isolated. Real-time qPCR showed that resveratrol at a concentration of 27.5 μM decreased virus production by 42% (Figure 5). Resveratrol at aconcentration of 55 μM reduced the production of EBV particles by 74% (Figure 5).

Previous studies showed that lytic EBV proteins actually induce the expression of B-cell growth factor, IL-6, cellular IL-10 and viral IL-10, allowing B cells to grow more efficiently [17,18]. Lytically-infected cells also produce VEGF and thus contribute to angiogenesis in both B-cell and epithelial-cell malignancies [18]. Therefore, new treatment strategies aimed at completely suppressing the expression of all lytic viral proteins is usefully in controlling early EBV-associated malignancies. This study finds that resveratrol significantly reduces the expression of EBV immediate-early proteins, Rta, Zta and EA-D in a dose-dependent manner (Figure 2 and Figure 3). In other words, resveratrol interferes with an early step of EBV replication cycle. The concentration of resveratrol needed to inhibit EBV immediate-early proteins expression by 50% (EC_50_) is approximately 24 μM. The cytotoxicity of resveratrol was determined by the MTT assay and resveratrol under the concentration of 55 μM did not affect the P3HR1 cells viability. Earlier study showed that EGCG inhibits the expression of EBV lytic proteins at concentration 50 μM [11]. The concentration of glycyrrhizic acid required to inhibit EBV EA/VCA expression by 50% is 42 μM [19]. These result suggested that resveratrol is more effective than EGCG and glycyrrhizic acid in inhibiting the expression of EBV immediate-early genes. 

Our study also demonstrates that resveratrol inhibits the transcriptional activity of BRLF1 and BZLF1 promoters, in turn, affect viral lytic proteins expression. Moreover, the inhibition actually decreases the production of mature viral particles. Real-time qPCR indicates that the effective concentration of resveratrol required to inhibit EBV genome copy numbers by 50% (EC_50_) is 52.2 μM (Figure 5). The EC_50 _values of resveratrol obtained by real-time qPCR is approximately 52.2 μM, in contrast to the 24 μM value determined by flow cytometry. However, the inhibitory profile remains unchanged, indicating that flow cytometry is as reliable as the real-time qPCR method for initial drug screening. 

The molecular mechanism underlying the inhibition of EBV early gene expression by resveratrol is unclear. Previous studies showed that resveratrol inhibits the activation of p38 MAPK, ERK and JNK signaling and affects the activation of the promoters that are activated by AP-1 or ATF2 [20]. Because both the BRLF1 and BZLF1 promoters are strongly activated by AP-1 and ATF2 [21,22,23]. The inhibition of the activation of signaling pathways may explain how resveratrol inhibits the EBV lytic cycle. Resveratrol also has been reported to activate the deacetylase activity of sirtuin protein [24]. Histone acetylation at the BRLF1 promoter allows the virus to express Rta and to activate the viral lytic cycle [25]. Moreover, Zta-directed nucleosome acetylation activates the transcriptional activity of viral immediate-early gene [26]. Thus, the inhibition of resveratrol on deacetylase activity of sirtuin protein may involve in the transcriptional inhibition of BRLF1 and BZLF1 promoters.

## 3. Experimental

### 3.1. Compound

Resveratrol (Sigma, St. Louis, MO, USA) was dissolved in dimethylsulfoxide (DMSO) before use.

### 3.2. Cell culture and lytic induction of EBV

P3HR1, a Burkitt’s lymphoma cell line which is latently infected by EBV, was cultured in RPMI 1640 medium containing 10% fetal calf serum (Biological Industries, Beit Haemek, Israel). Cells were treated with 3 mM of sodium butyrate (SB) to induce the EBV lytic cycle [27]. 

### 3.3. Cell viability assay

A 1 mg/mL solution of [3-(4,5-dimethyldiazol-2-yl)-2,5-diphenyltetrazolium bromide (MTT) in RPMI 1640 medium was added to 1 × 10^5 ^P3HR1 cells. The dehydrogenase activity of the viable cells was measured using the method of Carmichael *et al. *[28].

### 3.4. Indirect immunofluorescence staining

Immunofluoresence staining was performed as described [12], using mouse monoclonal anti-Rta, anti-Zta (Argene, Varilhes, France), and anti-EA-D antibodies (Chemicon, Temecula, CA, USA). Secondary antibodies used in the study included Alexa Fluor 488-conjugated goat anti-mouse IgG from Invitrogen. DNA was visualized by staining with 4’,6’-diamido-2-phenylindole (DAPI). Cells were examined under the Axioskop 2 plus fluorescence microscope (Zeiss, Oberkochen, Germany). 

### 3.5. Flow cytometric analysis

P3HR1 cells were treated with antibodies as described for indirect immunofluorescence analysis and fixed in 4% paraformaldehyde. Finally, cells were resuspended in 1% paraformaldehyde and analyzed using a flow cytometer (model FACScanTO, BD Biosciences, New Jersey, USA).

### 3.6. Transient transfection and luciferase assays

P3HR1 cells (5 × 10^6^) were transfected with 10 μg of pRLUC and pZLUC with a Bio-Rad electroporator using the method of Chang *et al. *[25,29]. Cells were harvested at 24 h after transfection and luciferase activity was determined using a luminometer (Berthold, Bad Wildbad, Germany) according to a method described elsewhere [25].

### 3.7. Quantification of EBV particles produced by P3HR1 cells

P3HR1 cells were cultured for 5 days. EBV particles released into the culture medium were harvested by centrifugation. Viral DNA was extracted and the amount of EBV DNA was determined by real-time PCR using an iCycleriQ multicolor real-time PCR detection system (BioRad, CA, USA) with primer and a probe that were specific to the EBNA1 gene [30]. The copy number of the EBV genome was calculated using maxi-EBV DNA extracted from *E. coli* as a reference. The molecular weight of maxi-EBV is about 1.2 × 10^7^ and 1 ng of maxi-EBV equals to 5.05 × 10^6^ copies of the maxi-EBV genome.

### 3.8. Statistical analysis

Data were analyzed statistically by one-way analysis of variance (ANOVA) using the SAS JMP 6.0 software package. Data are presented as means ± S.D. and a *p* value of < 0.05 was regarded as significant.

## 4. Conclusions

To summarize the whole study, our results clearly demonstrate that resveratrol inhibits the transcription of lytic genes and the lytic cycle of EBV to reduce the production of viral particles. Resveratrol could be of potential use for the development of anti-EBV drugs.
